# Understanding Users’ Experiences of a Novel Web-Based Cognitive Behavioral Therapy Platform for Depression and Anxiety: Qualitative Interviews From Pilot Trial Participants

**DOI:** 10.2196/46062

**Published:** 2023-06-20

**Authors:** Jane Shkel, Gavin Green, Stacey Le, Benjamin Kaveladze, Veronique Marcotte, Kevin Rushton, Theresa Nguyen, Stephen M Schueller

**Affiliations:** 1 Department of Psychological Science University of California, Irvine Irvine, CA United States; 2 Department of Medicine University of California, Irvine Irvine, CA United States; 3 Mental Health America Alexandria, VA United States

**Keywords:** anxiety, depression, cognitive behavioral therapy, clinical trial, intervention, qualitative research, digital mental health intervention

## Abstract

**Background:**

Digital mental health interventions (DMHIs) can help bridge the gap between the demand for mental health care and availability of treatment resources. The affordances of DMHIs have been proposed to overcome barriers to care such as accessibility, cost, and stigma. Despite these proposals, most evaluations of the DMHI focus on clinical effectiveness, with less consideration of users’ perspectives and experiences.

**Objective:**

We conducted a pilot randomized controlled trial of “Overcoming Thoughts,” a web-based platform that uses cognitive and behavioral principles to address depression and anxiety. The “Overcoming Thoughts” platform included 2 brief interventions—cognitive restructuring and behavioral experimentation. Users accessed either a version that included asynchronous interactions with other users (“crowdsourced” platform) or a completely self-guided version (control condition). We aimed to understand the users’ perspectives and experiences by conducting a subset of interviews during the follow-up period of the trial.

**Methods:**

We used purposive sampling to select a subset of trial participants based on group assignment (treatment and control) and symptom improvement (those who improved and those who did not on primary outcomes). We conducted semistructured interviews with 23 participants during the follow-up period that addressed acceptability, usability, and impact. We conducted a thematic analysis of the interviews until saturation was reached.

**Results:**

A total of 8 major themes were identified: possible opportunities to expand the platform; improvements in mental health because of using the platform; increased self-reflection skills; platform being more helpful for certain situations or domains; implementation of skills into users’ lives, even without direct platform use; increased coping skills because of using the platform; repetitiveness of platform exercises; and use pattern. Although no differences in themes were found among groups based on improvement status (all *P* values >.05, ranging from *.*12 to *.*86), there were 4 themes that differed based on conditions (*P* values from .01 to .046): helpfulness of self-reflection supported by an exercise summary (greater in control); aiding in slowing thoughts and feeling calmer (greater in control); overcoming patterns of avoidance (greater in control); and repetitiveness of content (greater in the intervention).

**Conclusions:**

We identified the different benefits that users perceived from a novel DMHI and opportunities to improve the platform. Interestingly, we did not note any differences in themes between those who improved and those who did not, but we did find some differences between those who received the control and intervention versions of the platform. Future research should continue to investigate users’ experiences with DMHIs to better understand the complex dynamics of their use and outcomes.

## Introduction

### Background

The prevalence of mental health disorders continues to increase at a rapid pace, with approximately 11.7% of US adults reporting symptoms of anxiety and 4.8% reporting symptoms of depression in 2021 [1]. Despite these high rates, a large proportion of individuals with mental health disorders do not receive treatment [2]. Barriers to receiving mental health care include cost, limited availability of treatment providers, transportation issues, long waitlists, stigma, and the fear of discrimination [3-6].

Most people (90%) in the United States are internet users [7]; therefore, using technology to develop and deploy mental health support is a scalable way to efficiently help people who might not otherwise receive mental health treatment. Digital mental health interventions (DMHIs), which are web-based or mobile interventions, can help mitigate such barriers by allowing people to privately access mental health care immediately within the context of their daily lives, usually for free or at a lower cost than traditional in-person therapy. In addition, the ability to access such interventions from home can help avoid childcare and transportation costs. DMHIs have been shown to be as effective as face-to-face therapy in treating mental health disorders [8,9]. As such, the popularity and use of DMHIs have gradually increased in the last decade [10].

However, despite the general effectiveness of DMHIs, not every person who uses them experiences benefits [11]. To date, most research on DMHIs has used quantitative measures and relied primarily on group averages to assess outcomes [12-15], subsequently masking individual differences. Therefore, understanding users’ varied experiences is a gap in the research. In addition, quantitative measures in clinical trials often focus on symptom reduction, which is not always the outcome that matters most to the participants [16]. For example, in one study of patients with depression and their caregivers and providers, outcomes such as motivation, functioning, social isolation, safety, social representation, and stigmatization were all identified as important [17]. We need opportunities for participants to provide feedback on traditional quantitative outcome measures to ensure that important outcomes are not missed.

Another important aspect to understand is participants’ experiences of a particular intervention—what they viewed as most helpful or what they disliked. Although engagement has been noted as a challenge in DMHI research and development [18,19], it is worth noting that mental health interventions overall often experience suboptimal engagement, as many patients drop out before receiving a full course of treatment [20,21]. Learning about users’ perspectives and incorporating their feedback into the design of interventions is central to improving them, as such improvements could increase initial engagement, adherence, and the potential impact on users [10]. Recent efforts have started to incorporate methodologies to involve and solicit end users in the early development and iterative evaluation, including leveraging methods from human-centered design [22,23]. Furthermore, it is worth noting that not everyone’s experiences will be the same. This refers to both clinical outcomes, as noted earlier, and people’s experiences of and perspectives on interventions. Providing opportunities for participants to speak more directly about their experiences through qualitative methods can help researchers delve deeper into the specific needs of individuals.

Some studies have used qualitative methods to evaluate the impact of DMHIs [24]. For example, some studies have examined people’s experiences of deployments of various DMHIs “in the wild,” such as cognitive behavioral therapy (CBT) platforms [25] or mood-tracking apps [26]. Other studies have examined people’s experiences with specific DMHIs, usually during the early development and evaluation. For example, Boucher et al [27] used a qualitative approach to determine how 11 adults experiencing loneliness reacted to Happify Health, which included specific tracks focused on defeating loneliness [27]. Qualitative methods have also been used to gain insight into medical students’ preferences for tailored DMHIs [28] and people’s general concerns and acceptability about using DMHIs for self-management of severe mental health problems [29].

Despite the current qualitative research that has been completed thus far, there continues to be a need for more work in this area, especially for studies evaluating the effects of specific DMHIs. A common challenge in such cases is trying to systematically include interviewees who (1) can provide a relatively even distribution of types of users and their perspectives on the DMHI (eg, perceptions of the DMHI from those who improved or did not improve from using it) and (2) has shared experiences of using the same DMHI around the same time period. However, many of the interviewees in qualitative studies are chosen on a first-come, first-served basis [27] or are self-selected users [30] who have all had different experiences with DMHIs (eg, free-range users or users from different time periods). In this study, we sought to include interviewees who all experienced the same DMHI and who we were able to group based on our knowledge of how they benefited from the intervention and which intervention they received.

### Objective

Specifically, this study aimed to understand the experiences of participants using a novel DMHI platform for depression and anxiety, “Overcoming Thoughts.” “Overcoming Thoughts” was based on CBT principles, which are commonly used in DMHIs for depression and anxiety [12]. Our investigation was focused on understanding users’ perceptions of the features of the platform, as well as their experience using the platform, including their own assessment of its impact on them. The interviewees were selected based on their group assignments and symptom improvement. This study was part of a broader evaluation of “Overcoming Thoughts,” but has relevance to both understanding the impact of digital CBT platforms on users and informing the design of future DMHI and digital CBT platforms.

## Methods

### Procedures

Participants were drawn from those who participated in a pilot randomized controlled trial (RCT) examining the “Overcoming Thoughts” web-based platform (ClinicalTrials.gov NCT0422 6742). “Overcoming Thoughts” is based on CBT and consisted of 2 exercises involving cognitive restructuring and behavioral experimentation practices. In the pilot RCT, participants were asked to use the platform for 8 weeks. Participants completed trial assessments at baseline, midtreatment (4 weeks), posttreatment (8 weeks), and follow-up (16 weeks) time points. The RCT compared 2 versions of this platform: a version with crowdsourced support (ie, allowing users to engage with peers’ content in structured ways) and a self-guided control (ie, working through the platform’s exercises alone without seeing others’ responses). We found preliminary evidence that participants in both groups improved on the Depression Anxiety and Stress Scale (DASS) after 8 weeks of using the “Overcoming Thoughts” platform, with no significant differences between the crowdsource and control groups [31].

Recruitment for the pilot RCT was conducted on the Mental Health America screening website [32]. Recruitment advertisements were placed on the screener results to visit a short eligible screener. Participants who had elevated levels of depression and anxiety on the Patient Health Questionnaire-9 (PHQ-9) [33] or Generalized Anxiety Disorder-7 (GAD-7) [34], defined as PHQ-9 or GAD-7 scores >9, completed a baseline assessment for full eligibility. Potential interviewees for the exit interviews were selected through purposive sampling by taking a random sample balancing across conditions (crowdsource vs control) and symptom improvement (improved vs did not improve). Symptom improvement was determined by users’ scores (change from baseline to week 8) on the DASS, with improvement defined as a symptom change of at least 25%. The selected participants were invited to participate in the optional interview via email. If they did not respond to the initial email invitation, 2 additional attempts were made before considering that they had declined the opportunity. Exit interviews were conducted at the postintervention time point (between the 8- and 16-week assessments) using a semistructured interview guide that addressed issues of acceptability, usability, and impact (Textbox S1 in Multimedia Appendix 1 presents the semistructured interview guide). Each interview lasted between 30 and 60 minutes and was conducted via Zoom (Zoom Communications Inc) by a research assistant who was a masters-level trainee and had been trained in the study procedures and interview protocol. The interviews were audio recorded and transcribed.

### Ethics Approval, Informed Consent, and Participation

The study procedures were approved by the Institutional Review Board at the University of California, Irvine (HS #2020-6071) as part of the pilot RCT. All participants provided signed consent to participate in all the trial procedures and were informed that they might be selected to participate in an optional interview. Participants who completed the interview were offered an additional US $20 in compensation, which did not impact their compensation for the overall trial. The study data were deidentified before the analysis.

### Participants

Of the 45 participants invited to the exit interviews at the postintervention time point, 23 (51%) participants completed them. Of the 23 participants, 15 (65%) were female, 7 (30%) were male, and 1 (4%) was identified as nonbinary or other. Furthermore, 74% (17/23) were White, 9% (2/23) were Asian, 9% (2/23) were African American, 4% (1/23) was of more than one race, and 4% (1/23) did not report race. The mean age across this subset of participants was 34 (SD 11.1) years. As mentioned, the participants were invited based on both their condition assignment (intervention vs control) and their improvement status (improve vs did not improve). The sample size was determined based on an initial goal to recruit about 25% of the total sample, with an enrollment target for the RCT of 100 and for the interviews of 25. However, we reviewed and coded interview transcripts throughout the process and continued recruitment until thematic saturation was reached [35], resulting in our final sample of 23 interview participants. Although we randomly selected participants to invite, as the completion rate was 51% (23/45), we had slight differences in completions, with 10 intervention participants and 13 control participants and 13 participants who improved and 10 who did not. Among the interview participants, the mean baseline DASS score was 68.9 (SD 26.04) and the score at week 8 was 50.08 (SD 30.34). Among those identified as improvers, the baseline DASS score was 63.4 (SD 20.68) and the score at week 8 was 21.8 (SD 7.63), whereas among those identified as nonimprovers, the baseline DASS score was 73.2 (SD 29.61) and the score at the week 8 was 71.8 (SD 21.54).

### Analysis

We conducted thematic analysis to identify the key themes discussed in our interviews. Our thematic analysis followed 6-stage process formulated by Braun and Clarke [36], including iterative steps to identify the data, create a codebook and identify themes, review themes, and present the data. In the initial phase, we conducted affinity diagramming to familiarize ourselves with the data and to identify the initial themes. We began this process after 15 participants had completed the interviews not only to provide our team with sufficient content to review and start coding but also to allow us to assess whether we had reached thematic saturation and could stop recruiting participants. Our team randomly selected 4 interview transcripts, and 4 members of our team read 2 transcripts, identified themes in those transcripts, presented the themes to the group, and discussed until consensus resulted in 15 initial themes. Subsequently, 2 members of our team took those themes and created an initial codebook with definitions for each code to identify common themes in the participants’ experiences of the platform. Two independent raters (JS and GG) coded the remaining interview transcripts, noting additional codes that were then discussed among the research team until thematic saturation was reached, which occurred after approximately 20 interviews. We interviewed another 3 participants to ensure that no new trends were identified and then stopped recruiting for the exit interviews. The final codebook consisted of 19 codes (Table S1 in Multimedia Appendix 1 provides the list of codes and definitions). The raters returned to all transcripts to code them using the final codebook. The raters had 88.1% general agreement in codes across all transcripts. In addition to our thematic analysis, we compared differences in the frequency of themes that occurred between the crowdsource and control conditions and those who improved and did not improve using chi-square difference tests.

## Results

### Overview

Out of the 19 themes, there were eight themes that we identified in most interviewees (ie, >50%): (1) improvement in mental health, (2) self-reflection skills, (3) engraining skills learned from the platform into habit, (4) coping skills, (5) situational benefits, (6) areas for improvement, (7) repetitiveness of platform exercises, and (8) use pattern. These major themes were separated into 2 categories: impact of the platform on the users and feedback on the platform itself. The impact of either working through the exercises alone (ie, control group) or seeing others’ responses (ie, crowdsource group) was also evaluated and categorized under feedback on the platform itself. Table 1 depicts the full breakdown of the remaining 11 identified themes (ie, those mentioned by <50% of interviewees). These major themes are outlined with examples of interviewees’ responses in subsequent paragraphs.

**Table 1:** Frequency of codes identified across participant interviews.

Theme and rank		Code	Total (n=23), n (%)	Intervention (n=10), n (%)	Control (n=13), n (%)
**Improvement in mental health**					
	1	Improvement in mental health	20 (87)	9 (90)	11 (85)
	2	Self-reflection skills	19 (83)	7 (70)	12 (92)
	3	Situational benefit	19 (83)	7 (70)	12 (92)
	4	Application of skills or building habits	17 (74)	9 (90)	8 (62)
	5	Coping skills	13 (57)	6 (60)	7 (54)
	6	Calming or slowing down	11 (48)	2 (20)	9 (69)
	7	Breaking things down	10 (43)	5 (50)	5 (38)
	8	Lack of motivation	6 (26)	3 (30)	3 (23)
	9	Overcome avoidance	6 (26)	0 (0)	6 (46)
**Feedback on platform**					
	1	Areas for improvement	23 (100)	10 (100)	13 (100)
	2	Impact of working alone (control)	13 (57)	N/A^a^	13 (100)
	3	Repetitiveness of platform exercises	13 (57)	8 (80)	5 (38)
	4	Use pattern	12 (52)	5 (50)	7 (54)
	5	Flexibility	11 (48)	5 (50)	6 (46)
	6	Simplicity	11 (48)	3 (30)	8 (62)
	7	Impact of seeing others’ responses (crowdsource)	10 (43)	10 (100)	N/A
	8	Access challenges	8 (35)	5 (50)	3 (23)
	9	Ability to look at answers	5 (22)	0 (0)	5 (38)
	10	Freedom of scheduling	5 (22)	4 (40)	1 (8)

^a^N/A: not applicable.

### Impact of the Platform on the Users

#### Overview

The first category of themes evaluated the impact of the platform on users. Overall, interviewees indicated that they experienced many and varied positive impacts from using the platform, such as perceived improvement in their mental health. In addition, many interviewees noted improvement in more specific areas related to mental health, such as increased self-reflection skills, coping skills, and the ability to apply skills learned from the platform into their daily lives (ie, even without the direct use of the platform). Interviewees also mentioned that there were specific contexts or situations in which the platform was especially helpful for them over others.

#### Improvement in Mental Health

Of the 23 interviewees, 20 (87%) perceived improvement in their mental health because of using the “Overcoming Thoughts” platform. Notably, there was a wide range in the timeline of improvement, with some users saying they experienced benefits immediately (eg, “I would say it was kind of immediate. Kind of like a cup of coffee is how I can compare it to” [P18]); after several weeks (eg, “I saw the most impact between 3-5 weeks” [P13]); or never (eg, “I think it does little to help for people with severe mental illness like me” [P11]). Several participants mentioned that the platform was particularly beneficial in helping them process their feelings. For example, 1 participant described it as follows:

I think it was immediately, as soon as I used it the first day, that I saw the impact. That I was able to process what was happening with my emotions. I realized I have this tool now to help out and I can use it whenever I’m not feeling all that great.

P17

Similarly, another participant said the following:

I think the main impact was forcing me to process feelings. Probably at two weeks was when I realized, “Oh I guess I should actually figure out what I’m feeling,” and at least these prompts were helping me to do that since I don’t tend to do that. It was nice to have these prompts because again, I’m very bad at processing it on my own.

P16

Other participants said that the platform benefited their mental health by helping them to instill healthier thought patterns by regularly completing the platform’s exercises, supporting them when dealing with loneliness, or making it feel like having a friend to talk to.

#### Self-reflection Skills

Of the 23 interviewees, 19 (83%) noted that using the “Overcoming Thoughts” platform assisted in the process of reflecting on their thoughts, feelings, and emotions. Participants attributed the platform’s effectiveness at improving self-reflection skills to its structure and the way the prompts in the exercises were set up:

It built in a sort of process or procedure or SOP of evaluating my state of mind.

P2

Others compared using the platform to “thinking out loud” or writing in a diary or journal, which helped them look at their thoughts and feelings more in depth.

In addition, participants often described how the platform helped them shift their perspective and look at their thoughts or feelings from a different angle. For example, 1 participant said the following:

Helped to remind me to check in and think about the actual thoughts I was having instead of just kind of lingering in them. It gave me room to kind of step outside and see things from a different perspective.

P14

Furthermore, this change in perspective allowed some participants to reflect on and realize how their thoughts could be negatively impacting them:

What it showed me as I was typing it out, I was kind of recognizing my thoughts, seeing myself in this really low-level way and how I was talking so harshly, which was just eye-opening.

P19

I was more able to analyze myself and how I cope with things. I realized I was having a very bad attitude. Every little thing would complicate my whole day. I was thinking very negatively about everything.

P9

Although most participants referenced improvements in their self-reflection skills in general, some participants identified that the platform helped them reflect on specific situations or problems they were experiencing. Examples included “dating and how I could improve myself” (P18) or helping with graduate school:

I’m a grad student so when I was having a rough time in my lab, I pulled it up and did the Overcoming Thoughts and mental processes activity. I was able to see the situation a little more clearly and the steps I needed to take to rectify the situation. It helped to put it into perspective.

P15

#### Application of Skills or Building Habits

Of the 23 interviewees, 17 (74%) noted that they were able to apply skills into their lives because of the learning stemming from the platform. In other words, they were able to internalize and mentally “go through” the platform’s activities, even without directly logging in and using the platform. For example:

When struggling with thoughts or self-doubt, whether it was just in my head, I found myself asking a lot of the types of questions that the prompts in activities would ask me as they came up in real life.

P1

Other participants commonly reported similar experiences:

I’d remember the questions and I’d do it in my head. Started changing my way of thinking after three weeks of using it.

P9

I started practicing doing the online prompts in my head more. If I’m worrying about something a lot or something is bugging me, I’d think, “Okay, what’s a different way of thinking about this? What can I do about it?” Allowed me to think about it even without getting on the website to participate but just by thinking about it. I learned how to apply coping mechanism for everyday use even without the tool.

P8

As a result, participants commonly reported feeling less dependent on the platform as their new skills developed into habits. Although their reliance on the direct use of the platform faded, they continued to reap the mental health benefits of learning stemming from the platform:

It helped me learn how to deal with my emotions more on my own. I could use it but I feel like I don’t need to now when I get upset. I can talk myself through it. I really like that it taught me something lasting.

P7

I’d say those first couple of weeks were enough and it instilled the thought patterns and habits in me. I was sort of seeking a way to help build better habits anyway.

P2

It felt like the natural flow of the tool became internalized and I could go through it without accessing the tool directly. Almost felt like it turned into a habit after a while of using it so I had less need for actually using it or logging in.

P3

By contrast, a few participants mentioned that they felt they needed more practice using the platform before it could become fully engrained into habit:

Has helped somewhat with new situations and stressors but need to go back and practice more for it to become more like auto pilot.

P5

#### Coping Skills

Of the 23 interviewees, 13 (57%) stated that they developed internal skills to help cope with stress, anxiety, and depression using the platform. One of the most mentioned coping skills learned was reframing thoughts into “less harsh” thoughts (P3). For example, some participants said the following:

It has helped me reframe my thinking where before, I would spiral downwards with my anxiety and thoughts. Now I’m able to kind of stop it in the beginning and reframe. I think it’s had a very positive impact.

P5

Helped me with the way I was thinking or how I can change one negative thought to a positive one, or take an unrealistic thought and think about it more in a more realistic way.

P9

Participants also reported other coping skills that the platform helped with, such as learning how to process their negative thoughts in general, talking themselves down when overthinking, and understanding which concrete steps or activities could help reduce their feelings of anxiety in the moment:

I only have one coping skill for my anxiety and it’s playing my guitar. The tool helped connect this together for me.

P17

#### Situational Benefits

Of the 23 interviewees, 19 (83%) indicated that there were certain times or domains when the “Overcoming Thoughts” platform was more helpful to them than others. In other words, the platform’s relative effectiveness depended on which situation they used it in. For example, 1 participant said that the platform helped more when feeling anxious rather than depressed:

My anxiety is easier to break down a bit and my depression feels like a big fat brick wall.

P17

Participants also commonly reported that the time of day also impacted how beneficial the platform was for them, with most people noting that it was more helpful in the morning:

When I did it in the morning or earlier in the day, it helped me focus less on the negative things the rest of the day. Versus when I did it at night, it was still nice, but I just went to sleep right after.

P16

Participants also commented on specific situations in which the platform was particularly helpful for them:

I was trying to get the COVID vaccine 1.5 months ago and I was having all these fears about it, thinking about what if I get a rare allergic reaction or something happens to me. After a couple tries with the tool, I was able to get the vaccine and second dose and everything. It really helped me, and that was really the reason why I was able to get the vaccine.

P9

One of the days, I typed about how I got a B on an assignment and I was so upset about it. That was one of the times where the tool was really effective because it asks, “If this were true, what would that mean of you?” and that made me think about why it’s so important to me, why grades have become almost like a part of my identity. In terms of dealing with failure, it’s really where that tool shined a lot for me.

P19

Other situations in which participants noted that the platform was particularly helpful included job-related stress, coping with loss, physical appearance, interpersonal relationships, road rage incidents while driving, loneliness, and improving productivity.

Conversely, participants also referenced contexts in which they felt that the platform was less effective for them. These included situations when participants felt that they were at their lowest points mental health–wise or when they felt less in control of the situation, such as in their relationships with others:

When I was at my lowest during the 8-week period, I didn’t want to use the tool. I didn’t want to do anything. It’s that state of mind when you’re so upset and so angry that you just kind of lose hope and you just kind of want to do nothing. I don’t know if there’s anything that made the program less effective, the reason I wouldn’t hop on when I was sad or angry was because when I’m in that space, I just don’t want to do anything. I don’t think any extra like glamour or better prompts would’ve helped me. I don’t think I even reached out to my therapist.

P19

I had been in a panic attack for two hours and I think I was so far into the attack I couldn’t pull myself out of it naturally even using the tool.

P13

Another situation that participants mentioned the platform felt less helpful for was when they felt general stress not tied to a discrete event:

[L]ess effective for generalized anxiety or having wild thoughts about things that aren’t connected directly to something.

P3

### Feedback on the Platform

In this second category of codes, participants provided feedback on the platform itself. The most common themes that occurred were areas for improvement, repetitiveness of the platform exercises, use pattern, and the different impacts of working alone (ie, for interviewees from the control group) or seeing others’ responses (ie, for interviewees from the crowdsource group).

#### Areas for Improvement

Of the 23 interviewees, 23 (100%) suggested ways for the “Overcoming Thoughts” platform to be expanded on, improved, or used in the future. One of the most common suggestions was to embed a reminder or notification system into the platform to encourage or “nudge” users to log on and use the platform more. Other ideas to improve the platform included turning it into a mobile app rather than a browser, incorporating breathing exercises, inserting short educational videos about various mental health topics, and adding a transcription option so that users could vocally dictate their thoughts rather than typing them out. Another suggestion was to expand the platform to teen users:

I know a lot of teens would find a lot of benefit in learning these skills early and how to form correct thoughts.

P23

Although the interactive (ie, crowdsourced) version of the platform allowed users to view others’ responses and click a “Like” or heart button on responses they related to, several participants wished for an even more interactive version of the platform. Suggestions included adding the ability to comment on others’ posts and engage in 1:1 chats or discussion forums with other users (eg, “like an open chat opportunity to connect with people experiencing a similar situation.” [P23]). Another participant explained:

I wish you could’ve interacted with other people more. Almost in a social media type of way. It would’ve been cool to establish friendships with people that get it. Because with depression, it’s kind of hard to open up to people that don’t get it. So there’s a little more safety and security with people that do understand it. Even if it’s just where you can post and comment and share support or commonality with other people’s posts.

P21

By contrast, a few participants recommended giving users the choice of whether they wanted others to see their responses, such as by having an on and off toggle button:

I feel like people should have the option if they want to do the peer-based one versus being alone. Some people do like input from others.

P7

#### Repetitiveness of Platform Exercises

Of the 23 interviewees, 13 (57%) indicated that the exercises on the platform started to feel repetitive, as the same set of 8 questions was displayed in the same order each time (Figure S1 in Multimedia Appendix 1 provides the list of questions for each activity). For example, one participant stated the following:

The questions never change. It’s the same questions over and over.

P11

Others referenced how the platform would feel more engaging if more variety was embedded into the activities:

I think for it to be something I’d want to use everyday, I would probably feel more motivated if I didn’t know exactly what I was going to be asked. So switching up the wording or just kind of giving it this variety.

P16

At the same time, others felt that the repetitiveness of the questions held some positive benefits, as it helped them understand how frequently some of their thoughts commonly recurred:

At first, I became a little frustrated or irritated because I was like, “Ah I’m inputting the same thing on a daily basis.” But eventually, it got to the point where I was making those connections of like, “You’re really having this thought everyday, let’s really tweak it.” So I feel like having it be a repetitive thing helped further implement the use of the positive thinking.

P23

#### Use Pattern

Of the 23 interviewees, 12 (52%) discussed aspects of the frequency with which they used the platform. Although many discussed their use, their reported frequencies displayed considerable variance. Some participants said they used the platform inconsistently in general (eg, “I didn’t use it every week because I would forget about it.” [P12]), at least once a week (eg, “I’d try to do the prompt all the way through at least once a week.” [P8]), or multiple times per week or sometimes even per day (eg, “I used it at minimum three times a week. Some of those days, I’d use it multiple times a day.” [P17]).

In addition, participants often referenced their use patterns depending on need:

I don’t think I set up a schedule for it or anything, I just logged in whenever I was reminded of it or if I felt like I needed a place to vent but didn’t want to bother anyone else in my life”

P14

Similarly, others specifically noted using the platform less frequently as their mental health improved:

In the beginning, I tried to use it 3-5 times a week. As it went on, I did less and less but it’s because I was doing better so I found myself having to check in less and less.

P15

#### Impact of Working Alone (Control)

A total of 57% (13/23) of interviewees participated in the control group. People in this group worked through the platform exercises alone without being able to view other users’ responses to the activities. Participants often referenced their personalities and differing comfort levels, with sharing as a common reason why they liked or disliked working through the platform privately. For example, some participants indicated their general discomfort when talking about sensitive topics (ie, mental health) or fear of judgment from others as a frequent reason why they preferred working alone:

I could see the benefits of having other users, but for me, I prefer to do stuff alone. I feel like if I were put into the one with other users, I would be worried they might be judgmental, it’s one of my anxieties. Preferred to work through activities by myself without worrying about other users.

P7

However, some participants felt that the platform was less engaging without a more interactive component and disliked working alone without external input:

It’s not the same as when you have somebody. It’s less engaging. It’s the nature of human beings. It probably would’ve helped to see others’ responses. It probably would make you dig a little bit deeper.

P20

#### Impact of Seeing Others’ Responses (Crowdsource)

A total of 43% (10/23) of interviewees belonged to the crowdsource group. People in this group were able to read and interact with other users’ responses to the exercises in structured ways (ie, by “liking” or relating to others’ responses). Several participants noted that it was helpful to see other people’s responses because they could obtain ideas and inspiration if they ever became stuck on a question:

There are times where I struggle trying to think of other things or how to respond. So, I’ll scroll through the comments and kind of get an idea and see how other people have kind of dealt with their things.

P6

Participants also commonly reported that seeing others’ responses helped them feel less alone, promoted a sense of community, and even motivated them to share more honestly about the experiences they were going through:

I really liked being able to see other people’s submissions. That almost made me feel less alone and even gave me ideas about what I wanted to talk about that day.

P19

By contrast, a few participants found other users’ submissions distracting and felt that it impeded their own progress with the platform’s activities:

Honestly, I would get distracted by other users’ responses. “Oh, if they’re on the fifth question and they’re already responding, I wonder what their initial problem was.” I started to focus less on my problem and what I was trying to accomplish and I started going down other rabbit holes.

P5

Another participant referenced how the quality of other users’ responses (ie, those that were “just filling this out to fill it out” rather than thinking thoughtfully about the platform’s questions) negatively impacted their experience:

When I see other comments where it’s just kind of like the response rather than actually trying to reshape thoughts into something positive, they’ll just continuously be negative. It just kind of makes me a little sad. Those comments don’t really affect me but it just kind of sucks to see that. I don’t usually go through comments, just when I am particularly having a hard time thinking of responses. But it’s like, “Alright, I guess this person’s just filling this out to fill it out.”

P6

Similar to the control group users, some crowdsource participants felt completely neutral. In such cases, participants said they did not notice other users’ responses at all or seeing them did not have much impact on them:

It didn’t really affect me much just because I didn’t have context for what they were saying so it really could’ve been anything.

P17

### Comparisons of Themes Across Improvement Status and Assigned Condition

In addition to the qualitative analysis, we conducted chi-square difference tests to determine whether the frequency of themes varied across the groupings that formed the basis of purposive sampling. Contrary to expectations, we found no significant differences in themes among interviewees based on their improvement status (all *P* values >.05, ranging from *χ*^2^_1_=2.4, *P*=.12 to *χ*^2^_1_=0.03, *P*=.86). In other words, whether the interviewees’ symptoms improved or did not improve on the DASS after the intervention did not impact the frequency of particular codes between the 2 groups.

However, we did find significant differences among the groups based on the treatment condition that participants were assigned to for 4 themes. Although both groups had access to this feature, more control users (5/13, 38% of interviewees) mentioned that they found the ability to look at a summary of their exercise responses helpful compared with 0% of crowdsource users (*χ*^2^_1_=4.9, *P*=.03). One control user described as follows:

Helped to look at summary at the end and realize how often that thought occurs for me. It was that accumulation and that gentle reflection back of “Oh wow, this thought is here a lot.”

P3

The control group (9/13, 69%) was also more likely than the crowdsource group (2/10, 20%) to report that the platform was helpful in slowing down their thoughts and feeling calmer (*χ*^2^_1_=5.5, *P*=.02). One control user mentioned as follows:

It made the process of trying to better a situation or calm myself down more clear, which it wasn’t before.

P15

In addition, interviewees from the control group (6/13, 46%) were more likely than the crowdsource interviewees (0%) to note that the platform helped them overcome patterns of avoidance (*χ*^2^_1_=6.2, *P*=.01).

The platform helped kick me into action to do things I’d been putting off because of emotions that may come up from taking care of that stuff.

P10

By contrast, more crowdsource participants (8/10, 80%) relative to control participants (5/13, 38%) indicated that they found the questions in the platform’s exercises to be repetitive (*χ*^2^_1_=4.0, *P*=.046). Interestingly, this was despite the crowdsource users having access to more content than the control users (ie, being able to see other users’ responses to the questions and having the “I Relate” button feature).

## Discussion

### Principal Findings

Our findings explored users’ perceptions of the “Overcoming Thoughts” platform. We found that users tended to discuss 3 main aspects of their experiences: the benefits they gained from using the platform, their perceptions of the characteristics of the platform, and their use of it. Users identified a range of perceived benefits including improvement in mental health, self-reflection, and coping skills. Furthermore, reflections on the platform itself identified opportunities for improvement and desired features that users would want on a digital CBT platform. One unexpected finding was the lack of differentiation of themes between those who improved and did not improve on the main clinical outcome. This is particularly noteworthy for themes related to users’ perceived benefits from the platform. These findings hold implications not only for our understanding of the “Overcoming Thoughts” platform but also for digital CBT and DMHIs more generally.

Our qualitative approach was able to identify a range of user-reported improvements beyond changes in depression, anxiety, and stress. Such an approach might be especially useful in an “experimental therapeutics” paradigm, as defined by the National Institute of Mental Health, which requires determination of target engagement in addition to assessing symptom change [37]. The potential mechanisms that we identified included self-reflection, improvement in coping skills, and the use of these skills in real-world settings. Prior conceptualizations of experimental therapeutics for DMHIs have focused on engagement as a potential target mechanism [38]. However, our findings suggest that engagement, if defined by the use of the DMHI platform throughout the intervention, may not sufficiently relate to benefits. Instead, many users noted that they were able to apply the platform’s skills to their lives even without the direct use of the platform. Although this was not explicitly promoted by the platform, engraining the learned skills into habit is a proposed mechanism through which CBT may have lasting benefits [39].

Even with digital platforms that, unlike therapists, can be used throughout one’s day, skill acquisition and application may be an important aspect of positive and lasting benefits. These findings might help explain the mixed literature regarding the relationship between treatment adherence to DMHI and clinical outcomes [40]. Our findings suggest that proposing and investigating specific proximal outcomes may be a useful complement to engagement in understanding the mechanisms of DMHIs. Users also mentioned the specific contexts in which they found the platform to be particularly helpful (eg, in the morning and when feeling anxious or depressed) or less effective (eg, when feeling at their lowest points and relationship issues). This information reveals that users did not view the platform as a one-size-fits-all resource for every situation they encountered. Instead, its relative perceived effectiveness seemed to depend on which context they used it in. This insight could be helpful for the development and deployment of similar DMHIs in the future, such as by matching certain clinical profiles to DMHIs or providing guidance to users on specific contexts or when to use the DMHI.

We also received important feedback on the platform itself, such as how often participants used it and potential features that users desired, for example, embedding a notification system to remind people to use the platform more often as well as creating more novelty and variability to avoid repetitiveness. Indeed, considering the basic design principles for DMHIs may be useful to support their efficacy even with reduced professional interaction. Although identifying design principles is more common in human-centered design literature [41] than in clinical science, some efforts to unpack design principles from learning theories have been made [42]. Specifically, Hitchcock et al [42] identified 3 principles from learning theories: repeated testing, interleaving and varying, and spacing. Although we predicted that our interactive “crowdsource” features would lead to better engagement, our participants also discussed some ways in which these features might be problematic for their use of the platform. For example, one interviewee suggested that it may be useful to give people the option (eg, an on and off toggle) to choose whether they want to work through the platform’s exercises privately or share their responses with others. Implementing such a change might lead to a more complicated user experience, but the important lesson we glean from this is that forcing such an interaction might be problematic for at least some users. Interestingly, we did not note any differences in themes among those who improved and did not improve on the primary outcome measure for the clinical trial, which was the reduction of DASS total scores. We should note that the participants who did not improve did start out with higher levels of depression, anxiety, and stress at baseline, which might reflect baseline differences between participants. Nevertheless, these participants who did not experience symptom improvement at the end of the study were just as likely to perceive mental health benefits and have similar perceptions of the platform as those whose symptoms did improve at the end of the study. Other studies have demonstrated that changes in symptom improvement on clinical measures do match participants’ self-ratings of changes in their mental health [43] and quality of life [44]. It is worth noting that we did not directly ask people whether they thought the platform improved their depression, anxiety, or stress. Instead, we asked people what they obtained out of using the platform or how they used any skills from the platform in their lives. We expected that those whose symptoms did not improve would have been less likely to perceive subjective mental health benefits or had more negative perceptions of the platform compared with those whose symptoms did improve, and vice versa, but this was not the case based on our qualitative analysis.

Although there were no differences between the participants who improved and those who did not improve, we did find some differences between the 2 conditions (control vs crowdsource). Control users were more likely than crowdsource users to comment on the helpfulness of the summary pages provided at the end of the platform’s exercise, as they allowed them to review and reflect on their previously submitted responses. Control users were also more likely to note perceived improvement in areas such as calmness and overcoming avoidance. By contrast, crowdsource users were more likely than control users to note that they found the platform’s exercises repetitive, despite crowdsource content adding more novelty through access to others’ responses. The specific reason for these differences between the 2 groups is unclear, but one contributing factor could be that the control users experienced less content in general. This may have allowed them to “turn inward” and focus more on observing and becoming more aware of their own thoughts and feelings, rather than focusing outward on external input from others and becoming “distracted” as one crowdsource user mentioned. It is possible that working more deeply through the platform helped the activities resonate more with the control users.

### Limitations

It is worth noting that some limitations of our study might have affected the findings. We collected insights on participants’ experiences using the “Overcoming Thoughts” platform, which is a novel DMHI and might not reflect experiences with DMHIs more broadly. Our intervention was meant to have brief interactions with a stronger focus on interactive exercises than on psychoeducation. As such, it may not be generalizable to platforms that have a more didactic format. Although we randomly selected 45 potential participants based on purposive sampling, we conducted only 23 interviews. As such, our interviewees might not reflect a representative sample of all the users of our platform. However, the purposive sampling method is also a strength such that we have roughly equivalent numbers of those who received the intervention and control conditions and those who improved and did not improve on our primary outcome measures. Although a total of 23 participants and at least 10 per group might be small, it is consistent with the recommendations for qualitative interviewing [45]. In particular, given our approach of purposive sampling, our sample was well contextualized, which provides greater information power for qualitative synthesis. It would be worthwhile to follow-up on some of our qualitative findings with quantitative research, perhaps using measures focused on skill acquisition and self-reflection as potential mechanisms for such an intervention.

### Conclusions

This study explored users’ perspectives on a novel DMHI based on cognitive behavioral principles. Our purposive sampling method, selecting a relatively even distribution of those who improved and did not improve on the main clinical outcome, as well as those who received the intervention (crowdsource) and control (self-guided) conditions, allowed us to explore differences in themes across different benefit categories and conditions. Surprisingly, we found no significant differences in themes between those who improved and those who did not improve on the primary outcome measures of depression, anxiety, and stress. However, we did find several differences in themes between those who received the crowdsourced and the self-guided version of the platform. Although we designed the crowdsourced version with the hope that it would drive engagement and benefit, we identified some challenges with the version that demonstrated how such features could potentially hinder people’s comfort with and use of the platform (eg, worries about others judging their responses, privacy reasons, and discomfort talking about sensitive topics like mental health). Providing users with the option to either share their responses with others or keep them private may be a useful feature for similar DMHIs in the future.

We also identified some potential benefits of such a platform, which might be useful targets to continue to explore in future investigations of DMHIs more generally. For example, our findings revealed that most interviewees (17/23, 74%) noted that they were able to apply strategies learned through the platform into their lives even without the direct use of the platform. In other words, they described their newly learned CBT-based skills as eventually becoming engrained into habit. Consequently, they continued to experience benefits from the skills learned through the platform, even though they logged onto the platform less frequently as time went on. It is important to appreciate that not all uses are equal. Having clear target uses for DMHI platforms and metrics to evaluate their use is a beneficial way to move development and evaluation forward. This might lead to the better use of A/B testing and iterative data-driven approaches to improve DMHIs.

Our qualitative investigation into users’ perceptions of our “Overcoming Thoughts” DMHI revealed valuable insights into the nuanced dynamics of the perceived benefits and characteristics of the platform. As the use of CBT-based DMHIs continues to increase, understanding the varied experiences of users is critical for the continued development and improvement of such DMHIs to maximize their impact.

## Appendix

Multimedia Appendix 1 Semistructured interview guide, final codebook, and questions contained within the Overcoming Thoughts platform.

## Abbreviations

CBT: cognitive behavioral therapy
DASS: Depression Anxiety and Stress Scale
DMHI: digital mental health intervention
GAD-7: Generalized Anxiety Disorder-7
PHQ-9: Patient Health Questionnaire-9
RCT: randomized controlled trial

## Data Availability

Quantitative data reported in this study are available from the National Institute of Mental Health Data Archive (NDA). The NDA is a collaborative informatics system created by the National Institutes of Health to provide a national resource to support and accelerate research on mental health. Data set identifiers: Collection ID #3219, National Institute of Mental Health Data Archive Digital Object Identifier 10.15154/1528642. Qualitative data are not available, as participants did not consent for the interview data to be shared publicly.
