# Supplementary material for: Understanding Users’ Experiences of a Novel Web-Based Cognitive Behavioral Therapy Platform for Depression and Anxiety: Qualitative Interviews From Pilot Trial Participants
Source: JMIR Form Res. 2023 Jun 20;7:e46062. doi: 10.2196/46062 (PMC10337366; doi:10.2196/46062)
Supplement: Multimedia Appendix 1 [file formative_v7i1e46062_app1.docx]

**Supplemental Materials**

### Textbox S1. Semi-structured interview guide for exit interviews.

We want to learn more about your experience using the Overcoming Thoughts tool, and ways that it may have been helpful or unhelpful. During this call, you are the expert, and there are no right or wrong answers. We want to make a platform that is as useful as possible, so any feedback you have is greatly appreciated.

What did you like about this tool? Why? (Expansion Questions: Think less about the design of the tool. Think more about the content of the activities and how they helped with emotions or skills)

Tell me about a time when using the tool went really well. What happened and why would you say that went well?

How many times/how long (# of weeks?) did you have to use the tool before seeing an impact? What type of impact did you see?

Now that you’ve used it for 8 weeks, what have you gotten from the use of this tool?

From what you learned online in this tool, did you use any of these skills in your real life? If so, what parts did you apply? How?

What other life experiences could something like this help you with?

Crowdsource: How did seeing others’ responses affect you? / Control: How did it feel to work through these activities alone/without other users’ input?

What did you dislike about this tool? Why? (Think less about the design of the tool. Think more about the content of the activities and how they helped with emotions or skills)

Tell me about a time using the platform that didn’t go well. What happened and why would you say that it didn’t go well?

Would you choose this platform over other platforms available? Why or why not?

Is this platform worth continuing to invest in? If yes, what are the parts we should focus on most?

What else do you want me to tell me about this tool that I haven’t asked yet?

### Table S1. Final codebook, primary codes, subcodes, and definitions.

| **Primary Code** | **Subcodes** | **Definition** |
| --- | --- | --- |
| *Primary codes represent common concepts within the data* | *Subcodes represent variations of the primary code. For example, the primary code "benefit" indicates that the subject mentioned benefiting from using the platform. The subcodes indicate the varying times at which benefit occurred, such as "Immediately" or "at two weeks"* | *Code definitions as agreed upon by coders* |
| Ability to Look at Answers | Positive, Negative | **PRIMARY CODE:** User mentioned the impact of reviewing previous submissions **SUBCODE**: specifically how this feature influenced them |
| Access Challenges |  | User indicated challenges to accessing the platform, such as login error or difficulty finding the platform |
| Applying Skills/Building Habits |  | Refers to applying skills in one's life as a result of learning stemming from the platform |
| Benefit | Immediate, 2 weeks, 3-5 weeks, 4-5 weeks, 2-3 weeks | **PRIMARY CODE:** User indicated an improvement in their mental health, as a result of using this platform. **SUBCODE:** The time at which the user noticed this improvement during the 8 week trial |
| Break Things Down |  | This platform assisted the user in breaking down their thoughts and experiences |
| Broader Implications |  | User suggests how this platform may be expanded on/improved/used in the future |
| Calming / Slowing Down |  | This platform assisted the user in slowing down or feeling calmer |
| Coping Skills |  | Refers to the development of internal skills to help cope with stress, anxiety, and depression THROUGH THE USE OF THE PLATFORM |
| Flexibility |  | User indicated appreciating being able to approach using the tool in different ways, either through choosing between one of the two preset routes, just looking at other users responses, or other alternative use cases |
| Freedom of Scheduling |  | Respondent indicated appreciating the freedom to choose when and how much to use the tool |
| Impact of Seeing Others' Responses | Positive, Neutral, Negative | **PRIMARY CODE:** User mentioned the ability to see others' responses **SECONDARY CODE:** How they felt toward this feature |
| Impact of Working Alone | Positive, Negative, Neutral | **PRIMARY CODE:** User mentioned the experience of working through platform alone/without seeing others' responses **SUBCODE:** How they felt about working through activities alone |
| Lack of Motivation |  | User struggled with motivation to start or fully complete a session using the tool at some point during the trial (e.g., due to depression/anxiety symptoms) |
| Overcome Avoidance |  | Use of this platform allowed the user to overcome patterns of avoidance |
| Repetitive |  | User indicated that the activities on the platform were repetitive |
| Self-Reflection |  | Refers to the platform itself assisting in the process of reflecting on one’s thoughts, feelings, and emotions |
| Simplicity Tech |  | The user liked the simplicity of the platform |
| Situational Benefit |  | The user indicated that there were times or domains where this platform was more helpful to them than others |
| Use Pattern | Weekly, Daily, Couple times per week | **PRIMARY CODE:** User indicates the frequency at which they used the platform **SUBCODE:** Specifics of frequency |

### Figure S1. Display of questions for each activity.

**What thought are you struggling with right now?**

**Focus on your thoughts** and practice more flexible thinking.

**Explore Thoughts to Feel Better >>**

**Focus on your actions** and consider different things you could do.

**Take steps to feel better >>**

Let’s rework the thought you struggle with. What is a healthier version of your thought above? This might be a different way of viewing the situation or a more compassionate statement to tell yourself.

What evidence in your life supports your reworked thought above?

What core belief is driving your initial thought above? (If you are struggling to identify it, try asking yourself, “If this were true, what would it mean about me? Why does this matter to me? What would happen if this were true? What would happen next?”)

Rework the core belief. What is a healthier version of your core belief?

What evidence in your life supports this healthier core belief?

Why is it important for you to let go of this thought? OR what would letting go of this thought let you do?

Pretend you’re talking to a friend. What would you say to encourage them to recognize and rework thoughts like you do above?

What good would come from reflecting on your thought and reworking it like you did above?

What could you do to make you feel better or think differently than the thought above?

What success might come from doing the thing above?

What is one obstacle you may encounter if you do the thing above?

How will you deal with that obstacle you identified to do the thing above?

What would be the first step of doing the thing above?

What would you need to help you do the thing above?

Pretend you’re talking to a friend. What would you say to encourage them to do things despite thoughts, like you did above.

What good would come from reflecting on your thought and reflecting on doing something to think and feel better like you did above?
